# Screening of new British thraustochytrids isolates for docosahexaenoic acid (DHA) production

**DOI:** 10.1007/s10811-017-1149-8

**Published:** 2017-04-30

**Authors:** Loris Fossier Marchan, Kim J. Lee Chang, Peter D. Nichols, Jane L. Polglase, Wilfrid J. Mitchell, Tony Gutierrez

**Affiliations:** 1 Institute of Mechanical, Process & Energy Engineering, School of Engineering and Physical Sciences, Heriot-Watt University, Edinburgh, EH14 4AS UK; 2 CSIRO Oceans and Atmosphere, GPO Box 1538, Hobart, TAS 7001 Australia; 3 Institute of Life and Earth Sciences, School of Energy, Geoscience, Infrastructure and Society, Heriot-Watt University, Edinburgh, EH14 4AS UK; 4 Institute of Biological Chemistry, Biophysics and Bioengineering, School of Engineering and Physical Sciences, Heriot-Watt University, Edinburgh, EH14 4AS UK

**Keywords:** Thraustochytrid, Docosahexaenoic acid, Omega-3 fatty acids, Biodiesel, By-product

## Abstract

Thraustochytrids isolated from hot tropical and sub-tropical waters have been well studied for DHA and biodiesel production in the last decades. However, little research has been performed on the oils of cold water thraustochytrids, in particular from the North Sea region. In this study, thraustochytrid strains from British waters showed high relative levels of omega-3 long-chain (≥C_20_) polyunsaturated fatty acids (LC-PUFA), including docosahexaenoic acid (DHA, 22:6ω3). The relative levels of DHA (as % of total fatty acids, TFA) in the different British strains are hitherto amongst the highest recorded from any thraustochytrid screening study, with strain TL18 reaching up to 67% DHA in modified Glucose-Yeast Extract-Peptone (GYP) medium. At this screening stage, low final biomass and fatty acid yield were observed in modified GYP and MarChiquita-Brain Heart Broth (MCBHB), while PUFA profiles (as % of PUFA) remained unaltered regardless of the culture medium used. Hence, optimizing the medium and culture conditions to improve growth and lipid content, without impacting the relative percentage of DHA, has the potential to increase the final DHA concentration. With this in mind, three strains were identified as promising organisms for the production of DHA. In the context of possible future industrial exploitation involving a winterization step, we investigated the recycling of the residual oil for biodiesel use. To do this, a mathematical model was used to assess the intrinsic properties of the by-product oil. The results showed the feasibility of producing primary DHA-rich oil, assuming optimized conditions, while using the by-product oil for biodiesel use.

## Introduction

Thraustochytrids are heterotrophic heterokonts or stramenopiles, certain strains of which are recognized for their ability to produce substantial amounts of omega-3 fatty acids, a high percentage of which is docosahexaenoic acid (DHA) (Lewis et al. 1999; Lee Chang et al. 2014). DHA is a major structural lipid which can be found as a constituent of phospholipids or triacylglycerols, or as free fatty acids in animals. It also has a key role in cell signalling, cell interaction and membrane fluidity (Colomer et al. 2007). DHA plays an important role in human health, in particular during the early stage of foetal and post-natal neuronal, retinal and immune system development (Swanson et al. 2012). There are positive correlations between DHA consumption and reduced disease, particularly in the prevention of cancers and cardiovascular diseases, improvement of inflammation response systems and maintenance of brain and learning functions (Horrocks and Yeo 1999; Lee Chang et al. 2013b). This knowledge has led to a growing demand for omega-3 supplemented food and other products. In fish, DHA has also been reported as essential for the normal growth and development of the animal, thus making it an indispensable component in aquaculture feeds (Sargent et al. 1999).

Currently, the production of omega-3-rich oils for human and animal diets relies primarily on wild-harvested fish and other marine animals such as krill (Pike and Jackson 2010). Paradoxically, this makes the expansion of the aquaculture industry directly limited by the global fishing capacity and its supply of DHA. However, with global fishing capacity reaching maximum levels since the early 1990s, aquaculture has overtaken the traditional wild-harvest fishing industry to meet the demand for fish and has become the fastest growing animal food-producing sector (FAO 2014). Hence, the supply of wild fish, and therefore omega-3 oils, is becoming tighter, emphasizing the need for alternate, renewable and sustainable sources of DHA (Pike and Jackson 2010). To meet this demand, the potential of thraustochytrids as DHA producers has been recognized (Barclay et al. 1994), and some examples of the omega-3 oils they produce have since been developed into commercially available bio-ingredients, such as Life’s DHA from DSM and DHAid from Lonza (Ratledge 2012). Nevertheless, further work is still needed to improve DHA yields from thraustochytrids, including the isolation and screening of new strains, together with their optimization using established or new fermentation strategies for production and the investigation of new substrates for even more cost-effective thraustochytrid growth.

In addition to the growing volume of research and development on the use of thraustochytrid oil in human and animal nutrition, there is also interest in the potential of these organisms for producing biodiesel (Lee Chang et al. 2012). With global economies depending almost entirely on fossil fuel resources, and taking into account the global depletion of natural reserves and the inextricable link between fossil fuel use and global warming, there is a need for sustainable alternatives to fossil fuel (Tietenberg and Lewis 2009). As a result, biodiesel has been promoted as an alternative to diesel fuel (European Commission 2003; Energy Policy Act 2005), and regulations for standardization of biodiesel were introduced, such as European standard EN 14214 and the ASTM D6751 in the USA and Canada. Since field crop-based biodiesel is associated with major socio-economic and environmental limitations, research focusing on single-cell oil-based biodiesel, including from thraustochytrids, has expanded (Meng et al. 2009). A major limitation to the implementation of thraustochytrid-derived oil is that the production of fish- or crop-derived oils remains more cost-effective, as a short-term application.

Most of the research on DHA from thraustochytrids to date has been performed with sub-tropical strains, many of which have been isolated from Malaysian, Japanese and Chinese coastal waters (Fan et al. 2002; Li et al. 2009; Byreddy et al. 2015; Chen et al. 2015; Manikan et al. 2015). Other work has been done on isolates from temperate regions such as southern Australian waters and cold temperate waters of Canadian littoral (Burja et al. 2006; Lee Chang et al. 2012). Here, we present the first detailed investigation on the oil composition and content of several new thraustochytrid strains isolated from Scottish waters. We investigated the DHA content and the qualitative properties of fatty acid profiles of these strains, with a view for potential applications in animal and human nutrition. We also performed a mathematical analysis based on the assumption that a winterization or other separation/fractionation step could be used for the purification of the DHA-rich oil, in order to investigate the potential use of the remaining by-product oil for biodiesel application.

## Materials and methods

### Thraustochytrid strains, maintenance and inoculum preparation

The thraustochytrid strains used in the present study are part of a unique British collection of strains isolated from lesser octopuses (*Eledone cirrhosa*), with a fatal ulcerative dermal necrosis at GPS coordinates 56°10′N 2°45′W, from within the lairs in which the octopuses lived, and from rainbow trout (*Oncorhynchus mykiss*), at GPS coordinates 56°26′N 5°50′W, also with skin lesions (Polglase 1980, 1981). Briefly, flasks containing 20 mL of sterile seawater were baited with sterile pine (*Pinus montana*) pollen. Flasks were then inoculated with a swab from a skin lesion or with 0.5 mL of water taken from the octopus lair. Thraustochytrid growth on the pollen grains was examined after 7, 10, 14, 21 and 28 days, and colonized pollen grains were used to inoculate agar plates (2% pre-soaked Fadenagar in seawater, 0.2% bacteriological agar (Difco, Germany), 0.03% peptone, 0.003% yeast extract (Difco, Germany) and 0.3% malt extract (Biomaltz, Germany). The plates also contained penicillin (50 to 100 IU mL^−1^) and streptomycin (100 μg mL^−1^). From these, small colonies were taken to inoculate further pollen-baited flasks. A glass micro-loop was then used to separate a pollen grain bearing a single thraustochytrid, which was used to establish an initial pure culture in a seawater and pine pollen culture flask.

Flask cultures were kept at 7, 10 or 15 °C and sub-cultured approximately every 6 months until the beginning of the present study, when colonized pollen grains were plated on MCBHB agar (Rosa et al. 2011) and single colonies were isolated and transferred into MCBHB medium in flask culture. One further strain (SM01) from soil in a salt marsh (56°02′N 2°58′W) was isolated similarly on pine pollen grains and MC agar (Rosa et al. 2011) in 2015 and subsequently maintained in MCBHB culture medium. The strain *Sicyoidochytrium* sp. NBRC 102979, obtained from the National Biological Resource Centre (NBRC) culture collection of the National Institute of Technology and Evaluation (NITE, Japan), was revived in H agar and medium (Honda et al. 1998) in half-strength seawater, before being treated and maintained in the same condition as the British isolates on MCBHB. This strain, isolated from Japanese sub-tropical waters, was selected to compare its performance with isolates from cold northern European waters. Inocula were prepared from axenic cultures maintained in MCBHB, with 1 mL of culture transferred to 20 mL (5% *v*/*v*) MCBHB or modified GYP (depending on the type of medium to be used for lipid analysis) in static pharmacological glass culture flasks and incubated at 20 °C for 2 weeks.

### Culture conditions for lipid analysis

The fatty acid profiles for each strain were evaluated in MCBHB, as previously described by Rosa et al. (2011), as well as using a modified version of the GYP media of Lee Chang et al. (2013a) (2% glucose, 0.2% peptone, 0.5% yeast extract, 0.2% monosodium glutamate, 0.1% corn steep liquor and 1 mL of each metal solution and vitamin solution as defined by the authors). Artificial sea salts used in both of these media were replaced by a 1:1 mixture of natural seawater/distilled water. Each strain was inoculated at 3% (*v*/*v*) into six 250-mL conical flasks for each medium and incubated on a rotary shaker (21 °C, 100 rpm). Optical density was measured at 600 nm using a Genesys 20 spectrophotometer (ThermoScientific, UK). At the end of exponential growth phase, three of the six flasks were terminated; the remaining three flasks were incubated for a further 4 days to reach late stationary phase. Cells were centrifuged (2600*×g* for 10 min) in 50-mL Falcon tubes and washed twice with 20 mL of 0.01 M phosphate buffered saline (PBS) at pH 7.2. Cell pellets were stored at −20 °C and then freeze-dried for subsequent fatty acid analyses.

### Direct transesterification of biomass for fatty acid methyl ester production

Freeze-dried cell pellets were directly transesterified to produce fatty acid methyl esters (FAME) as described by Lee Chang et al. (2011). Freeze-dried pellets were transferred into tared capped Pyrex test tubes and weighed. To each tube, 3 mL of methanol/chloroform/hydrochloric acid (10:1:1, *v*/*v*/*v*) was added, and then the tubes were heated to 80 °C in a water bath for 1 h. After cooling to room temperature, 1 mL of MilliQ water was added, and the mixture was extracted by the addition of 2 mL of hexane/chloroform (4:1). After vortexing and centrifugation at 400*×g* for 5 min, the upper non-aqueous layer of each sample was transferred to a clean test tube using a glass Pasteur pipette. Each mixture was extracted three times, and the combined extracted phases were then concentrated by blowing down under a stream of nitrogen gas until the volume was approximately 1 mL. The mixture of FAME was then transferred to a 2-mL amber glass vial (Agilent Technologies, UK) for subsequent fatty acid analysis.

### Determination of fatty acids profile

FAME were analysed using gas chromatography (GC) to determine fatty acid composition, as described by Lee Chang et al. (2014). For this, samples were blown down under nitrogen gas to remove residual solvent. A known volume of dichloromethane containing the internal injection standard (19:0 FAME) was then added to each sample vial. GC was performed on an Agilent Technologies 7890A GC (USA) equipped with a non-polar Equity-1 fused silica capillary column (15 m × 0.1 mm i.d., 0.1 mm film thickness), flame ionization detector and split/splitless injector. Samples were injected in splitless mode at an oven temperature of 120 °C, and after injection, the oven temperature was increased to 270 °C at 10 °C min^−1^ and then to 310 °C at 5 °C min^−1^. Peaks were quantified with Agilent Technologies ChemStation software (USA).

Confirmation of component identification was performed by GC-mass spectrometry (GC-MS) on a ThermoScientific 1310 GC coupled with a TSQ triple quadrupole. Samples were injected using a Tripleplus RSH autosampler into a non-polar HP-5 Ultra 2 bonded-phase column (50 m × 0.32 mm i.d. × 0.17 μm film thickness). The HP-5 column was of similar polarity to the column used for GC analyses. The initial oven temperature of 45 °C was held for 1 min, followed by temperature increase at 30 °C per min to 140 °C, then at 3 °C per min to 310 °C which was held for 12 min. Helium was used as the carrier gas. Mass spectrometer operating conditions were: electron impact energy 70 eV, emission current 250 μA, transfer line 310 °C, source temperature 240 °C, scan rate 0.8 scan s^−1^ and mass range 40–650 Da. Mass spectra were acquired and processed with Thermo Scientific XcaliburTM software (USA). Results are presented as the mean of area percent (%), mg g^−1^ biomass ± standard deviation (SD) and as mg L^−1^ of culture. All samples were prepared in triplicate.

### Determination of quality of the microbial oil for biodiesel production

Qualitative properties of the samples for potential use as biodiesel were determined according to the fatty acid profiles obtained from cells grown to the exponential phase in MCBHB medium followed by assuming a winterization or other process (Table 4), leading to the removal of LC-PUFA. This mathematical analysis was carried out to investigate potential use of the oil by-product remaining after the purification of the thraustochytrid crude oil, primarily aimed at production of omega-3 for commercial application. The saponification value (SV), iodine value (IV) and cetane number (CN) were calculated using the following Eqs. (1), (2) and (3), respectively, as described in Francisco et al. (2010):

1 $$ \mathrm{SV}=\sum 560 N/ M $$

2 $$ \mathrm{IV}=\sum 245 DN/ M $$

3 $$ \mathrm{CN}=46.3+\left(5458/\mathrm{SV}\right)-\left(0.255\times \mathrm{IV}\right) $$

where *N* is the percentage of each fatty acid component, *D* is the number of double bonds and *M* is the molecular weight (amu) of the FAME. The molecular weights of FAME used were listed by Møller (2011).

The degree of unsaturation (DU), the long-chain saturation factor (LCSF) and the cold filter plugging point (CFPP) were estimated by Eqs. (4), (5) and (6) (Ramos et al. 2009):

4 $$ \mathrm{DU}=\mathrm{MUFA}+\left(2\times \mathrm{PUFA}\right) $$

5 $$ \mathrm{LCSF}=\left(0.1\times 16\kern-0.23em :\kern-0.23em 0\right)+\left(0.5\times 18\kern-0.23em :\kern-0.23em 0\right)+\left(1\times 20\kern-0.23em :\kern-0.23em 0\right)+\left(1.5\times 22\kern-0.23em :\kern-0.23em 0\right)+\left(2\times 24\kern-0.23em :\kern-0.23em 0\right) $$

6 $$ \mathrm{CFPP}\ \left({}^{{}^{\circ}} C\right)=\left(3.1417\times \mathrm{LCSF}\right)-16.477 $$

where MUFA and PUFA are the percentages of the monounsaturated fatty acids and polyunsaturated fatty acids, respectively, and 16:0, 18:0, 20:0, 22:0 and 24:0 are the percentage for these respective fatty acids.

## Results and discussion

### Effect of growth phase and medium on fatty acid composition, final biomass and total fatty acid yields

**Fig. 1:**
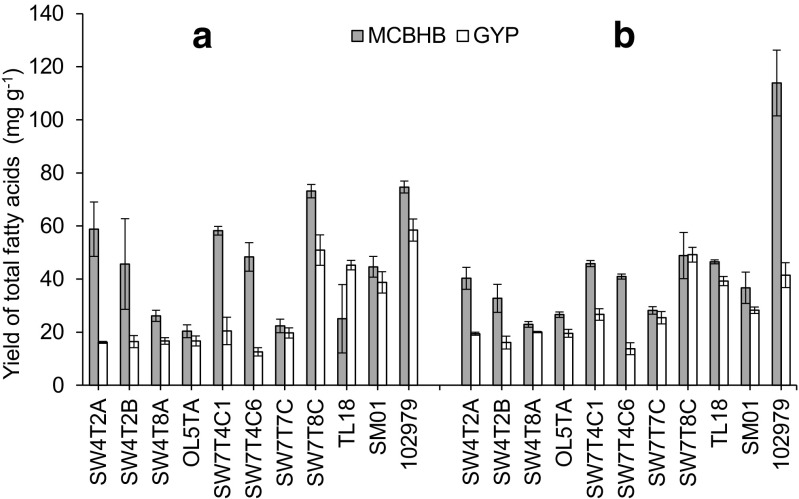
Fatty acid yields (mg g^−1^ of dry cell weight) of ten British isolates and reference strain *Sicyoidochytrium* sp. (NBRC 102979) grown in MCBHB (*grey bar*) and modified GYP (*open bar*) media at the **a** end of the exponential phase and **b** late stationary phase. Values represent mean ± standard deviation (*n* = 3)

The growth phase very clearly impacted the fatty acid composition (expressed as % of TFA) in both MCBHB and modified GYP media, as shown in Tables 1 and 2, respectively. In general, the relative levels of PUFA in both media increased during the stationary phase, while the proportion of saturated fatty acids (SFA) and monounsaturated fatty acids (MUFA) decreased. When grown in MCBHB medium, only strain SW7T8C showed a slight increase in the proportion of SFA and MUFA in the stationary phase, from 38.2 to 40.6% and 1.8 to 2.5% of TFA, respectively. In thraustochytrids, the biosynthetic pathways involved in the production of PUFA are the conventional fatty acid synthase route alongside that for polyketide synthase (PKS). The former is considered as the aerobic pathway and involves elongation of the carbon chain and formation of double bonds through a succession of elongase and desaturase enzymes (Ratledge 2004). In contrast, the PKS is sometimes described as the anaerobic pathway, and although not yet fully understood, it involves a “block building” system using the condensing enzyme 3-ketoacyl synthase among others. In both cases, LC-PUFA synthesis uses SFA and MUFA as precursors (Metz et al. 2001). Therefore, whichever of these pathways is primarily used by these microorganisms, the synthesis of LC-PUFA as an end product over time will result in a decrease of the precursors, which was observed as lower relative levels of SFA and MUFA and higher levels of LC-PUFA in the stationary phase of growth. In addition, the increase of PUFA in the stationary phase of growth can be partially explained by the decreased total fatty acid yields in the same phase by almost all strains (Fig. 1). This can result in higher relative levels of PUFA due to lower oil content. However, this is not true for all strains, such as the strains SW7T7C and *Sicyoidochytrium* sp. which showed both higher total fatty acid yields and relative levels of PUFA in stationary phase.

**Table 1:** Fatty acid composition (as percentage of total fatty acids) of ten British thraustochytrid isolates and reference strain *Sicyoidochytrium* sp. (NBRC 102979) grown in MCBHB medium in shake flask culture and harvested at the end of the exponential phase and late stationary phase. Values represent mean ± standard deviation (*n* = 3)

Strain	SW4T2A		SW4T2B		SW4T8A		OL5TA		SW7T4C1		SW7T4C6		SW7T7C		SW7T8C		TL18		SM01		102979	
Growth stage	Exp	Stat	Exp	Stat	Exp	Stat	Exp	Stat	Exp	Stat	Exp	Stat	Exp	Stat	Exp	Stat	Exp	Stat	Exp	Stat	Exp	Stat
Fatty acids																						
14:0	2.5 ± 0.4	1.1 ± 0.2	1.9 ± 1.1	0.7 ± 0.1	0.8 ± 0.3	0.7 ± 0.1	1 ± 0.3	0.5 ± 0	2.3 ± 0.1	1.4 ± 0.2	2.8 ± 0.5	2.1 ± 0.2	3.2 ± 1.6	1.2 ± 0.6	5.7 ± 0.6	5.2 ± 0.5	5.6 ± 5	1.7 ± 0.2	0.4 ± 0	0.4 ± 0.3	0.9 ± 0.1	0.5 ± 0.1
15:0	14.7 ± 3.6	10 ± 1.2	5.7 ± 3.8	6.6 ± 1.4	16.1 ± 1.1	10.3 ± 0.4	11.3 ± 2.4	5.6 ± 0.1	9.7 ± 0.6	5 ± 0.8	14.4 ± 2.3	9.4 ± 0.5	17.3 ± 3.5	7.6 ± 1.7	19.8 ± 0.4	18.1 ± 3.9	9.6 ± 8	6.2 ± 0.4	10.2 ± 1.4	8.6 ± 1.9	4 ± 0.7	2.2 ± 0.1
16:0	6.5 ± 1.3	8.7 ± 1.5	13.2 ± 11.5	9.1 ± 1.8	5.4 ± 0.5	4.8 ± 0.2	5.4 ± 1.1	3.8 ± 0.4	16.5 ± 1.2	15.6 ± 1.1	12.3 ± 2.4	13.6 ± 0.8	12 ± 2	9.7 ± 2.3	9.4 ± 0.2	12.8 ± 2.3	21.1 ± 13.8	8.5 ± 0.2	5.9 ± 0.7	6.2 ± 1	37.3 ± 0.6	27.8 ± 1.4
17:0	4.2 ± 1.2	5 ± 0.5	9.5 ± 9.2	6.3 ± 1.3	5.4 ± 0.6	4.4 ± 0.2	4 ± 0.8	3.3 ± 0.1	6 ± 0.4	3.6 ± 0.4	6.3 ± 1.2	4.8 ± 0.2	5.5 ± 0.7	4.3 ± 0.2	3.2 ± 0.1	4.5 ± 1	8.2 ± 5.3	3.7 ± 0.3	9.8 ± 1.5	8.5 ± 1.4	1.9 ± 0.2	1.1 ± 0
18:1ω9c	0.9 ± 0.1	0.5 ± 0.1	1.1 ± 0.6	0.4 ± 0	0.9 ± 0.1	0.6 ± 0	1.6 ± 0.2	0.7 ± 0	0.5 ± 0.1	0.3 ± 0	0.7 ± 0.1	0.6 ± 0	1.6 ± 0.4	0.8 ± 0.3	1.1 ± 0	1.2 ± 0.2	1.2 ± 0.7	0.5 ± 0.1	0.4 ± 0.1	0.3 ± 0.1	0.8 ± 0.3	0.2 ± 0
18:1ω7c	1.7 ± 0.3	2.1 ± 0.4	4.7 ± 3.9	3.5 ± 0.7	1.5 ± 0.1	1.3 ± 0.1	4.9 ± 0.7	3.2 ± 0.3	0.6 ± 0	0.7 ± 0	0.6 ± 0.1	0.6 ± 0	1.5 ± 0.2	1.8 ± 0.8	0.8 ± 0	1.3 ± 0.2	1.4 ± 0.8	0.8 ± 0	1.8 ± 0.2	1.3 ± 0.3	2.6 ± 0.5	4.5 ± 0.1
20:4ω6 ARA	1.1 ± 0.1	1 ± 0	0.6 ± 0.4	0.6 ± 0.1	1.1 ± 0	1.1 ± 0	1.4 ± 0.1	1 ± 0	0.5 ± 0.1	0.6 ± 0	1.6 ± 0.1	1.4 ± 0	1.3 ± 0.2	1.2 ± 0.3	1.4 ± 0.1	1.2 ± 0.1	1.2 ± 0.4	1.1 ± 0	0.8 ± 0.1	0.8 ± 0.1	0.4 ± 0	0.4 ± 0
20:5ω3 EPA	1.4 ± 0.2	3.1 ± 0.2	2 ± 1.3	4 ± 0.2	3.2 ± 0	4.3 ± 0.2	2.9 ± 0.3	4.5 ± 0.1	2.5 ± 0.2	4.3 ± 0.3	3 ± 0.4	4.5 ± 0.2	2.7 ± 0.8	4.7 ± 0.5	2.1 ± 0.2	3.1 ± 0.6	3.3 ± 1.3	5.2 ± 0.3	2.4 ± 0.1	3.4 ± 0.2	2.1 ± 0.2	2.4 ± 0.1
22:5ω6 DPAω6	3.3 ± 0.1	4 ± 0	3.3 ± 1.3	4.4 ± 0.4	1.1 ± 0.2	1.7 ± 0.1	1.3 ± 0.3	1 ± 0.3	5.8 ± 0.1	7 ± 0.3	5.7 ± 0.5	6.7 ± 0.2	1.8 ± 0.3	1.4 ± 0	4.1 ± 0.1	4.2 ± 0.5	4 ± 1.6	6.2 ± 0.2	4.4 ± 0.1	4.8 ± 0.2	7.6 ± 0.4	1.5 ± 0.6
22:6ω3 DHA	46.8 ± 6.7	52 ± 3.8	47.3 ± 18.2	55 ± 4.3	51.2 ± 2.8	58.5 ± 1	48.5 ± 6.2	63.2 ± 1.7	47.9 ± 1.6	54.5 ± 2.9	41.4 ± 6.4	47.1 ± 1	38.1 ± 7.9	54.3 ± 4.7	38.1 ± 0.9	37.2 ± 6.2	36.1 ± 16.6	60.7 ± 0.3	55.3 ± 3.7	57.4 ± 5	30 ± 1.9	52.1 ± 2.3
22:4ω6 DTA	0.4 ± 0	0.2 ± 0.3	0.5 ± 0.3	0.9 ± 0.2	0.6 ± 0.1	0.8 ± 0	0.9 ± 0	0.8 ± 0.1	0.6 ± 0.1	0.7 ± 0	0.6 ± 0.7	0.8 ± 0.1	0.5 ± 0.1	0.8 ± 0.1	0.4 ± 0.3	0.5 ± 0.5	0.9 ± 0.7	0 ± 0	0.9 ± 0.1	0.8 ± 0.7	0.4 ± 0	0.4 ± 0
22:5ω3 DPAω3	1.2 ± 0.2	1.8 ± 0.3	1.1 ± 0.8	2.5 ± 0.3	1.1 ± 0.1	1 ± 0.1	0.9 ± 0.1	1.4 ± 0.1	0.9 ± 0.6	1.6 ± 0.1	0.9 ± 0.6	1 ± 0.6	0.9 ± 0.1	1.5 ± 0.1	1.2 ± 0.4	1.7 ± 0.1	1.6 ± 0.7	2 ± 0.1	1.1 ± 0.1	1.5 ± 1.6	0.8 ± 0	1.0 ± 0
Sum SFA	27.9 ± 4	24.7 ± 1.9	30.3 ± 15.2	22.7 ± 2.7	27.7 ± 1.4	20.2 ± 0.5	21.7 ± 2.8	13.2 ± 0.4	34.5 ± 1.4	25.7 ± 1.4	35.7 ± 3.6	29.9 ± 0.9	38 ± 4.4	22.8 ± 3	38.2 ± 0.8	40.6 ± 4.7	44.6 ± 17.5	20.1 ± 0.5	26.3 ± 2.1	23.7 ± 2.6	44.1 ± 0.9	31.6 ± 1.4
Sum MUFA	2.6 ± 0.3	2.6 ± 0.4	5.8 ± 4	3.8 ± 0.7	2.4 ± 0.2	1.9 ± 0.1	6.5 ± 0.7	3.9 ± 0.3	1 ± 0.1	1 ± 0.1	1.3 ± 0.2	1.2 ± 0.1	3 ± 0.4	2.6 ± 0.9	1.8 ± 0.1	2.5 ± 0.3	2.6 ± 1	1.3 ± 0.1	2.1 ± 0.2	1.6 ± 0.3	3.4 ± 0.6	4.8 ± 0.1
Sum PUFA	54.3 ± 6.7	62 ± 3.8	54.7 ± 18.3	67.5 ± 4.3	58.4 ± 2.8	67.4 ± 1	55.9 ± 6.2	71.8 ± 1.7	58.3 ± 1.7	68.6 ± 2.9	53.3 ± 6.5	61.4 ± 1.2	45.4 ± 7.9	64 ± 4.7	47.3 ± 1	47.9 ± 6.2	47.2 ± 16.8	75.1 ± 0.5	64.9 ± 3.7	68.6 ± 5.4	41.3 ± 2	57.9 ± 2.4
Sum ω3	49.4 ± 6.7	56.8 ± 3.8	50.4 ± 18.2	61.5 ± 4.3	55.5 ± 2.8	63.8 ± 1	52.3 ± 6.2	69 ± 1.7	51.3 ± 1.7	60.3 ± 2.9	45.3 ± 6.4	52.5 ± 1.2	41.7 ± 7.9	60.6 ± 4.7	41.5 ± 1	42 ± 6.2	41 ± 16.7	67.8 ± 0.4	58.8 ± 3.7	62.3 ± 5.3	32.9 ± 1.9	55.5 ± 2.3
Sum ω6	4.9 ± 0.1	5.2 ± 0.3	4.3 ± 1.3	5.9 ± 0.4	2.9 ± 0.2	3.6 ± 0.1	3.6 ± 0.3	2.8 ± 0.3	7 ± 0.1	8.3 ± 0.3	7.9 ± 0.9	8.9 ± 0.2	3.7 ± 0.3	3.4 ± 0.1	5.9 ± 0.3	5.9 ± 0.7	6.2 ± 1.7	7.3 ± 0.2	6.1 ± 0.1	6.3 ± 0.7	8.3 ± 0.4	2.3 ± 0.6

*ARA* arachidonic acid, *EPA* eicosapentaenoic acid, *DPAω6* docosapentaenoic acid (also termed Osbond acid), *DHA* docosahexaenoic acid, *DTA* docosatetraenoic acid, *DPAω3* docosapentaenoic acid (also termed Clupanodonic acid), *Exp* end of exponential phase, *Stat* late stationary phase

**Table 2:** Fatty acid composition (as percentage of total fatty acids) of ten British thraustochytrid isolates and reference strain *Sicyoidochytrium* sp. (NBRC 102979) grown in modified GYP medium in shake flask culture and harvested at the end of the exponential phase and late stationary phase. Values represent mean ± standard deviation (*n* = 3)

Strains	SW4T2A		SW4T2B		SW4T8A		OL5TA		SW7T4C1		SW7T4C6		SW7T7C		SW7T8C		TL18		SM01		102979	
Growth stage	Exp	Stat	Exp	Stat	Exp	Stat	Exp	Stat	Exp	Stat	Exp	Stat	Exp	Stat	Exp	Stat	Exp	Stat	Exp	Stat	Exp	Stat
Fatty acids																						
14:0	3.3 ± 0.1	1.9 ± 0.1	1.6 ± 0.7	1.2 ± 0.2	1.7 ± 0.3	1.2 ± 0	0.7 ± 0.1	0.6 ± 0	1.3 ± 0.3	0.6 ± 0.1	2.2 ± 0.6	1.2 ± 0.1	1.6 ± 0.2	0.6 ± 0.2	0.6 ± 0.1	0.5 ± 0	0.5 ± 0.2	0.4 ± 0	0.5 ± 0.1	0.6 ± 0.1	1.4 ± 0	0.8 ± 0.1
15:0	17.7 ± 1.3	11.8 ± 0.2	13.7 ± 5.4	1.4 ± 3.1	11.4 ± 0.7	8.5 ± 0.1	9 ± 1.6	6.4 ± 0.3	10.1 ± 2.3	5.9 ± 0.3	8.4 ± 5.9	2.2 ± 3.7	18.9 ± 3.9	6.9 ± 1.2	6 ± 0.5	5.7 ± 0.5	4.3 ± 3.8	3.8 ± 3.3	4.3 ± 3.7	6.3 ± 0.2	2.2 ± 0.2	1.7 ± 0
16:0	15.2 ± 0.6	11.9 ± 0.5	10.7 ± 2.4	10.2 ± 2.2	8.6 ± 0.8	7.6 ± 0.1	8.1 ± 0.8	7 ± 0.8	7.1 ± 1.9	4.4 ± 0.6	14.1 ± 3.4	10.6 ± 0.9	9.2 ± 0.3	6.5 ± 0.7	4.7 ± 0.8	4.8 ± 0.2	5.4 ± 0.5	5.6 ± 0.4	4.1 ± 0.1	4.1 ± 0.1	58.4 ± 2.4	27.4 ± 0.6
17:0	5.8 ± 0.4	4.4 ± 0.2	6.4 ± 1.8	4.8 ± 0.9	3.2 ± 0.2	2.9 ± 0.1	4.3 ± 0.5	2.9 ± 0.1	4.2 ± 1.1	2.7 ± 0.2	5.8 ± 1.6	3.4 ± 0.2	9 ± 1.5	4.4 ± 0.6	2.6 ± 0.3	2.8 ± 0.1	3.5 ± 0.1	3.4 ± 0.1	2.7 ± 0.1	2.6 ± 0.1	0.7 ± 0	0.3 ± 0
18:1ω9c	1 ± 0	0.6 ± 0	1 ± 0.4	0.6 ± 0.2	0.7 ± 0.1	0.5 ± 0	0.5 ± 0.1	0.4 ± 0	0.6 ± 0.1	0.2 ± 0	1.4 ± 0.4	0.8 ± 0.1	1 ± 0.1	0.5 ± 0.1	0.3 ± 0	0.2 ± 0	0.3 ± 0	0.3 ± 0	0.3 ± 0	0.3 ± 0	2.7 ± 1.9	0.1 ± 0
18:1ω7c	4 ± 0.1	2.6 ± 0.2	4.2 ± 0.8	2.2 ± 1.2	1.4 ± 0.2	0.8 ± 0.1	1.1 ± 0.1	0.8 ± 0.2	1.7 ± 0.4	0.4 ± 0.1	4.3 ± 2.9	4.6 ± 0.4	2.2 ± 0.1	1.4 ± 0.1	0.7 ± 0.1	0.8 ± 0.1	0.7 ± 0	0.7 ± 0.1	1.2 ± 0.1	1.2 ± 0.1	1.8 ± 0	2.6 ± 0.1
20:4ω6 ARA	0.8 ± 0	0.9 ± 0.1	1 ± 0.1	1.1 ± 0	0.9 ± 0	0.9 ± 0	0.8 ± 0.1	0.7 ± 0.1	1.1 ± 0.1	0.9 ± 0.1	1.1 ± 0.1	1.2 ± 0	0.8 ± 0	0.8 ± 0	1 ± 0.1	0.8 ± 0	0.9 ± 0	0.7 ± 0.1	1.1 ± 0	1 ± 0	0.2 ± 0	0.5 ± 0
20:5ω3 EPA	2.4 ± 0.1	3.4 ± 0.1	3.7 ± 1.3	5.4 ± 0.3	4.1 ± 0.2	4.9 ± 0.1	4.2 ± 0.7	4.2 ± 0.7	5.1 ± 0.5	7.5 ± 0.5	3 ± 0.5	4.5 ± 0.3	2.8 ± 0.4	4.7 ± 0.2	4.9 ± 0	5.1 ± 0.1	4.9 ± 0.1	4.9 ± 0.5	4.3 ± 0.1	4.9 ± 0.2	1 ± 0.1	2.4 ± 0
22:5ω6 DPAω6	2 ± 0.1	2.4 ± 0.2	2.5 ± 0.5	3.5 ± 0.2	3.5 ± 0.4	3.4 ± 0	2.4 ± 0.2	1.8 ± 0.4	3.5 ± 0.2	2.9 ± 0.7	2.3 ± 0.3	3.1 ± 0.4	2.4 ± 0.1	2.3 ± 0.5	1.5 ± 0.2	1.6 ± 0.2	1.5 ± 0.5	1.4 ± 0.4	1.4 ± 0.1	2.1 ± 0.5	4.1 ± 0.1	6.2 ± 1.1
22:6ω3 DHA	26.4 ± 2	39.8 ± 1.5	31.7 ± 9.9	46 ± 2.6	45.9 ± 2.6	52.4 ± 0.6	51.9 ± 3.6	60 ± 1.3	45.6 ± 7.4	58.9 ± 1.9	30.3 ± 7.1	44.8 ± 0.3	35.9 ± 5.9	57.1 ± 3.8	65.5 ± 0.5	65.9 ± 0.3	66.4 ± 2.3	66.9 ± 2	60.7 ± 2.1	58.2 ± 0.3	11.8 ± 0.4	51.6 ± 1.5
22:4ω6 DTA	0.5 ± 0	0.6 ± 0.1	0.7 ± 0.1	1 ± 0.1	0.9 ± 0	0.9 ± 0	0.7 ± 0.1	0.7 ± 0.1	0.8 ± 0.1	1 ± 0.1	0.7 ± 0.1	0.8 ± 0.1	0.6 ± 0	0.8 ± 0.1	0.6 ± 0.1	0.7 ± 0	0.7 ± 0.1	0.7 ± 0	0.5 ± 0	0.5 ± 0.1	0.2 ± 0	0.7 ± 0.1
22:5ω3 DPAω3	1.2 ± 0.1	1.7 ± 0.2	2.5 ± 0.9	4.1 ± 0.2	1.4 ± 0.1	1.7 ± 0.1	1.7 ± 0.4	1.8 ± 0.2	1.8 ± 0.2	2.6 ± 0.1	0.9 ± 0.1	1.4 ± 0.2	1.5 ± 0	2.2 ± 0	1.8 ± 0.1	2.1 ± 0.1	1.6 ± 0.1	1.7 ± 0.1	2 ± 0.3	2.2 ± 0.5	0.4 ± 0.1	1.2 ± 0.1
Sum SFA	42.1 ± 1.5	30 ± 0.6	32.4 ± 6.2	17.6 ± 3.9	25 ± 1.1	20.2 ± 0.2	22.1 ± 1.9	16.9 ± 0.8	22.7 ± 3.2	13.6 ± 0.6	30.4 ± 7	17.3 ± 3.8	38.6 ± 4.2	18.5 ± 1.5	13.9 ± 1.1	13.8 ± 0.5	13.7 ± 3.8	13.2 ± 3.4	11.6 ± 3.7	13.6 ± 0.3	62.7 ± 2.5	30.3 ± 0.6
Sum MUFA	5 ± 0.1	3.3 ± 0.2	5.1 ± 0.9	2.8 ± 1.3	2 ± 0.3	1.3 ± 0.1	1.6 ± 0.1	1.2 ± 0.2	2.3 ± 0.4	0.7 ± 0.1	5.7 ± 2.9	5.4 ± 0.4	3.2 ± 0.2	1.9 ± 0.1	1 ± 0.1	1 ± 0.1	1 ± 0	1 ± 0.1	1.5 ± 0.1	1.5 ± 0.1	4.5 ± 1.9	2.8 ± 0.1
Sum PUFA	33.2 ± 2	48.8 ± 1.5	42.2 ± 10	61.1 ± 2.7	56.8 ± 2.7	64.2 ± 0.6	61.8 ± 3.7	69.1 ± 1.6	58 ± 7.4	73.8 ± 2.1	38.3 ± 7.1	55.8 ± 0.6	44 ± 5.9	67.8 ± 3.8	75.4 ± 0.6	76.3 ± 0.4	76 ± 2.4	76.5 ± 2.1	70.1 ± 2.2	69 ± 0.8	17.7 ± 0.4	62.5 ± 1.9
Sum ω3	30 ± 2	44.9 ± 1.5	38 ± 10	55.5 ± 2.7	51.4 ± 2.7	59 ± 0.6	57.8 ± 3.7	65.9 ± 1.5	52.6 ± 7.4	69 ± 1.9	34.2 ± 7.1	50.7 ± 0.5	40.2 ± 5.9	64 ± 3.8	72.3 ± 0.6	73.1 ± 0.3	73 ± 2.3	73.6 ± 2	67 ± 2.2	65.3 ± 0.6	13.2 ± 0.4	55.1 ± 1.5
Sum ω6	3.2 ± 0.1	3.9 ± 0.2	4.2 ± 0.5	5.6 ± 0.2	5.4 ± 0.4	5.2 ± 0	4 ± 0.2	3.2 ± 0.4	5.5 ± 0.2	4.8 ± 0.7	4.1 ± 0.3	5.1 ± 0.4	3.7 ± 0.1	3.8 ± 0.5	3.1 ± 0.2	3.2 ± 0.2	3 ± 0.5	2.9 ± 0.4	3 ± 0.1	3.7 ± 0.5	4.5 ± 0.1	7.4 ± 1.1

Abbreviations: see Table 1

**Fig. 2:**
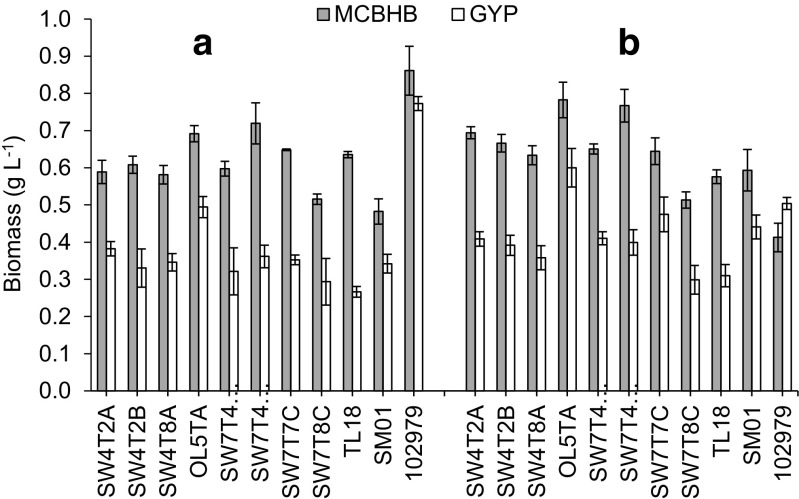
Biomass concentration (g L^−1^) of ten British isolates and reference strain *Sicyoidochytrium* sp. (NBRC 102979) grown in MCBHB (*grey bar*) and modified GYP (*open bar*) media at the **a** end of the exponential phase and **b** late stationary phase. Values represent mean ± standard deviation (*n* = 3)

Higher biomass concentrations (Fig. 2) and fatty acid yields (Fig. 1) were reached in MCBHB medium for all strains regardless of the growth phase, with the exception of *Sicyoidochytrium* sp. for which a higher biomass was observed in modified GYP at the late stationary phase of growth. However, in these screening media, the total lipid contents observed for the British isolates were low, ranging from 1.6 to 7.5% of dry cell weight regardless of the type of medium used. Hence, most of the fatty acids extracted are likely derived from membrane phospholipids rather than triacylglycerols. Similar results were obtained by Bowles et al. (1999), who showed that cold temperate strains (isolated from 59 to 61°N) only accumulated lipids to between 2.0 and 7.1% of dry cell weight, while cool temperate isolates (50–51°N) showed a lipid content between 1.6 and 13.6% of dry cell weight. Burja et al. (2006) also observed less than 10% of total lipid in dry cell weight biomass, for most of their isolates from cold Atlantic Canadian waters (from 40 to 50°N). For industrial applications, the total fatty acid yields must be improved to be commercially viable for use in the food and aquaculture industries. However, a higher glucose concentration (2% in modified GYP compared to 0.1% in MCBHB) did not induce a higher final biomass concentration, nor a higher accumulation of total lipids. This may be attributed either by the nutrient limitation in GYP (nitrogen or micronutrient insufficiency), or by an excess of glucose affecting growth in MCBHB.

Alternatively, the British thraustochytrids may be unable to utilize glucose and utilize another carbon source, such as amino acids. If this is the case, it implies that the higher biomass reached in MCBHB was due to other nutrients present such as peptones, yeast extract and brain heart broth that are complex nitrogen and carbon sources. Indeed, the total nitrogen and total carbon content in MCBHB were estimated to be 0.26 and 0.08% (ratio C/N equal to 0.3), respectively, whereas for modified GYP the total nitrogen content was estimated to be 0.11% and 0.8% for total carbon (ratio C/N equal to 7.45). Therefore, a higher nitrogen content and possibly a carbon source derived from amino acids were more beneficial for thraustochytrids growth than a high concentration of glucose.

Among the British isolates, the highest fatty acid yields were achieved in MCBHB by strain SW7T8C (73 mg g^−1^ DW) at the end of the exponential phase (Fig. 1), while the maximal biomass concentration at stationary phase of growth was found with strains OL5TA and SW7T4C6 (ca. 0.78 g L^−1^) (Fig. 2). Only strain TL18 showed higher fatty acid yield in modified GYP, which was achieved at the end of the exponential phase of growth (45.2 mg g^−1^ DW in modified GYP compared with 25.1 mg g^−1^ DW in MCBHB) (Fig. 1). Hence, under the growth conditions used in this study, MCBHB was generally found to be a more suitable growth medium because higher fatty acid yields and biomass concentrations were achieved. However, we note that in terms of yield, none of the strains out-performed *Sicyoidochytrium* sp. NBRC 102979. Again, similar final biomass concentrations were found by Bowles et al. (1999) and Burja et al. (2006) for strains isolated from other cold water environments. In both studies, a medium containing 0.5% glucose was used, and final biomass concentrations achieved ranged between 0.1 and 1.5 g L^−1^ during the initial screening stage. Therefore, the performance of the British strains was similar to strains that had been isolated from cold Atlantic waters, with similar biomass and total fatty acid yields obtained at the screening stage. Since lipid accumulation does not appear to be enhanced by an increased glucose concentration, an approach to achieving more competitive biomass concentration and total lipid yields may require development of an optimized MCBHB medium containing different nutrients such as complex carbon and nitrogen sources. Therefore, higher yields are also likely to be achieved, possibly by changing other aspects of the medium and/or growth conditions.

### Effect of medium on PUFA profiles

**Fig. 3:**
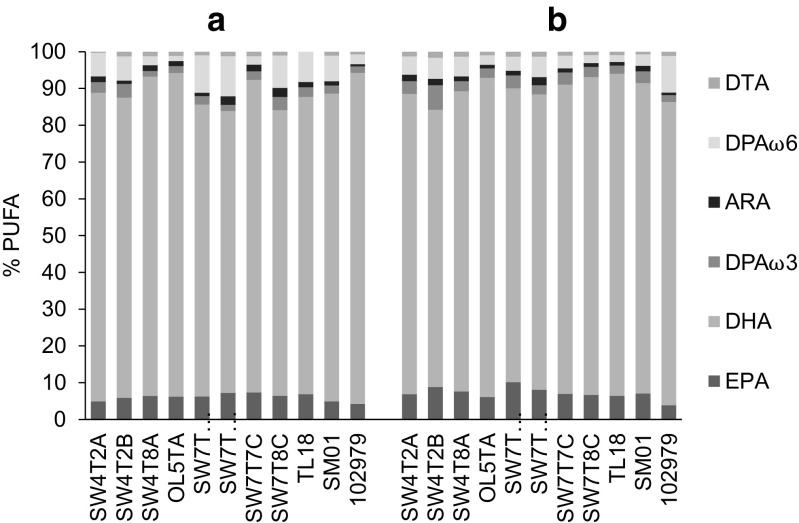
PUFA profiles expressed as % of total PUFA of ten British thraustochytrid isolates and reference strain *Sicyoidochytrium* sp. (NBRC 102979) grown in **a** MCBHB and **b** modified GYP medium in late stationary phase of growth. Values represent mean of independent triplicate (*n* = 3)

The total PUFA composition in the stationary phase across all the strains studied ranged from 47.9 to 75.1% of TFA in MCBHB medium (Table 1) and from 48.8 to 76.5% of TFA in modified GYP medium (Table 2). In MCBHB medium, the highest DHA levels of 63.2 and 60.7%, respectively, of the TFA were produced by strains OL5TA and TL18, while maximum DHA levels in modified GYP medium were produced by strains SW7T8C, TL18 and OL5TA at 66, 67 and 60% of the TFA. Overall, the medium type had little effect on the PUFA profile of the strains in the stationary phase of growth (Fig. 3); the production of omega-3 fatty acids prevailed over the production of omega-6 fatty acids in all cases, with DHA as the predominant PUFA component. The only exception was that a greater relative level of DPAω6 was observed for strains SW7T4C1 (10% of PUFA) and SW7T4C6 (11% of PUFA) in MCBHB medium (Fig. 3), with lower relative levels of DPAω6 produced in modified GYP (4 and 5% of PUFA, respectively).

Thus, the fatty acid composition of the British isolates showed a comparable PUFA profile in modified GYP and in MCBHB media, with a high DHA level (average 83% of total PUFA) and low levels of EPA (average 7% of total PUFA) and DPAω6 (average 5% of total PUFA). These results are in agreement with those of Huang et al. (2003) who observed no change in PUFA profiles of strains collected in coastal areas of Japan and Fiji when using different media containing different glucose concentrations. From these data, the British strains could potentially be of commercial interest for omega-3 oil production, as the DHA level (expressed as % TFA) is, to the best of our knowledge, among the highest recorded to date from an initial screening study. The highest level of DHA production by a thraustochytrid previously reported during a screening study was 61% of TFA by *Aurantiochytrium* sp. TC-39 (Lee Chang et al. 2012). As discussed below, further work to optimize DHA yields is warranted in order to explore the biotechnological potential of these strains.

### DHA concentrations

**Fig. 4:**
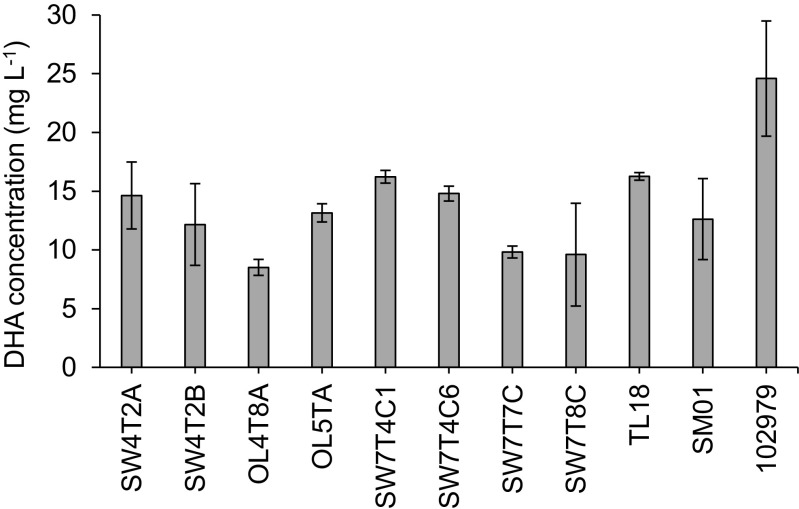
DHA content (mg L^−1^) of ten British isolates and reference strain *Sicyoidochytrium* sp. (NRBC 102979) grown in MCBHB medium in late stationary phase of growth. Values represent mean ± standard deviation (*n* = 3)

As described above, thraustochytrid strains grown in MCBHB medium showed higher fatty acid yields and biomass production compared to growth in modified GYP medium, while relative (% TFA) levels of PUFA were higher in the stationary phase. Therefore, the concentration of DHA in milligram per liter in MCBHB medium at stationary phase was examined. Among the British isolates, strains SW7T4C1 and TL18 showed the highest production with DHA concentration reaching 16 mg L^−1^ on average (Fig. 4). These yields were lower than for the reference strain *Sicyoidochytrium* sp. NBRC 102979 (24.6 mg L^−1^) and considerably lower than concentrations reported in other screening studies, with for instance *Aurantiochytrium mangrovei* FB3 which achieved a DHA concentration of 2.5 g L^−1^ (Fan et al. 2007). However, the low DHA concentrations achieved with the British strains can be mainly attributed to a low final biomass concentration reached in MCBHB. Indeed, when proportional levels of DHA were compared, the British strains exhibited the highest values (up to 63% DHA of TFA in MCBHB) (Table 3). These observations suggest that in a more suitable medium, higher levels of biomass and DHA concentrations could be achieved. For instance, for the organism *Aurantiochytrium* sp. TC-20, Lee Chang et al. (2012) reported a similar low DHA concentration (40 mg L^−1^), final biomass concentration (1 g L^−1^), total fatty acid content (7.8%) and a relatively high level of DHA (52% of TFA) during initial screening of this strain. Nonetheless, in subsequent studies with the same organism using further optimized culture conditions (fed-batch bioreactor with glycerol and varying nutritional supplements), the biomass reached 71 g L^−1^ and the DHA concentration was improved to 14 g L^−1^ of culture medium, while the relative proportion of DHA decreased slightly to 39% of TFA (Lee Chang et al. 2013a). Thus, it was demonstrated that it was possible to greatly increase the final biomass by optimizing culture conditions and culture strategy, while having only a moderate effect on the relative levels of DHA, hence allowing for considerable improvement in the culture DHA concentration. Taken collectively, the British strains show considerable potential for achieving higher DHA concentrations, due to their high relative levels of DHA coupled with the scope for improvement in final biomass concentrations and thereby fatty acid yields.

**Table 3:** Comparison of relative levels (as %TFA) and culture concentrations of DHA at the screening stage for different thraustochytrid strains

Strain	Location	Climate	DHA (% of TFA)	Fatty acids yield (mg g^−1^)	Biomass (g L^−1^)	DHA concentration (mg L^−1^)	Reference
*Aurantiochytrium* TC-20	North Queensland, Australia	Tropical	51.7	78	1	40	Lee Chang et al. (2012)
4-P-J	Hong Kong, China	Sub-tropical	31.0	–	15.6	–	Li et al. (2009)
*Aurantiochytrium mangrovei* FB3	Hong Kong, China	Sub-tropical	29.6	680.4	12.2	2457	Fan et al. (2007)
*Sicyoidochytrium* sp. NBRC 102979	Iriomote Island, Japan	Sub-tropical	52.1	113.9	0.41	24	This study
*Aurantiochytrium* TC-39	South East, Tasmania	Cool temperate	60.8	57	1.3	45	Lee Chang et al. (2012)
TL18	North Sea, Scotland	Cold temperate	60.7	46.5	0.58	16	This study
SW7T4C1	North Sea, Scotland	Cold temperate	54.5	45.8	0.65	16	This study
OL5TA	North Sea, Scotland	Cold temperate	63.2	26.6	0.78	13	This study

### Mathematical model to assess the by-product oil for use as biodiesel

To maximize the possible future industrial potential of the British thraustochytrid strains, a post-fermentation winterization or other separation/fractionation step was assumed. Briefly, the winterization (or other) process is a physical process that enables the separation of oils (generally composed of LC-PUFA) from fats (generally composed of SFA) according to their melting point by means of cooling and filtration. During the cooling step, SFA, which have higher melting points than LC-PUFA, will solidify first (stearin) while LC-PUFA will remain liquid (olein), thus allowing their separation and purification by subsequent filtration. Therefore, this process has the specific objective to increase the content of omega-3, and thus DHA, of the primary oil by removing SFA and MUFA. Meanwhile, this could potentially also enable the co-production of microbial biodiesel from the residual stearin (Lee Chang et al. 2013b). To assess this, relative levels of SFA and MUFA from cultures grown in MCBHB medium and harvested at the end of the exponential phase were re-calculated assuming that LC-PUFA were removed from the lipid phase by winterization, leaving MUFA and SFA as the predominant fatty acids (Table 4). Indeed, shorter chain (SC, ≤C_18_) fatty acids and a high degree of saturation are often seen as more suitable for optimal biodiesel properties (Lee Chang et al. 2013b).

**Table 4:** Theoretical fatty acid profile of oil derived from British thraustochytrids cultured in MCBHB medium and harvested at the exponential phase of growth followed by a theoretical winterization process. Values represent mean ± standard deviation (*n* = 3)

Strains	SW4T2A	SW4T2B	SW4T8A	OL5TA	SW7T4C1	SW7T4C6	SW7T7C	SW7T8C	TL18	SM01	102979
13:0	3.3 ± 0.2	0.3 ± 1.6	0 ± 1.7	0.1 ± 1.7	1.1 ± 0.2	2.5 ± 0.2	1.2 ± 1.7	7.1 ± 0.9	0.7 ± 1.2	0 ± 1.7	0 ± 0
14:0	5.9 ± 0.2	4.3 ± 0.7	2 ± 0.4	2.5 ± 0.3	5.7 ± 0.1	6.2 ± 0.2	6.2 ± 0.5	11.5 ± 0.1	10.8 ± 1	1 ± 0.1	1.5 ± 0.1
15:0	35.4 ± 0.3	12.9 ± 0.7	41.3 ± 0.1	27.7 ± 0.2	23.9 ± 0.1	32.5 ± 0.2	33.5 ± 0.3	39.8 ± 0	18.6 ± 0.9	29.7 ± 0.1	6.9 ± 0.2
16:1ω9c	0.1 ± 0.9	0.2 ± 1.2	1.4 ± 0.1	1.2 ± 1.2	0 ± 0	0.1 ± 0.9	0.3 ± 0.3	0.1 ± 1.7	0 ± 0	0 ± 0	0 ± 1.7
16:1ω7c	3.5 ± 0.2	1.9 ± 0.7	0.1 ± 0.9	1.6 ± 0.9	0.4 ± 0.2	1.2 ± 0.1	2.3 ± 0.3	2.3 ± 0	1.8 ± 0.7	0.6 ± 0.1	3.3 ± 0.2
16:0	15.6 ± 0.2	30.1 ± 0.9	13.8 ± 0.1	13.1 ± 0.2	40.6 ± 0.1	27.9 ± 0.2	23.3 ± 0.2	19 ± 0	40.7 ± 0.7	17.2 ± 0.1	64 ± 0
17:1ω8c	2.1 ± 0.2	1.7 ± 0.7	1 ± 0.1	1.9 ± 0.2	0.3 ± 0.2	1.1 ± 0.3	1.3 ± 0.4	2.3 ± 0.1	1 ± 1	1.2 ± 0.2	0.7 ± 0.3
17:1ω6c	2.6 ± 0.2	2.6 ± 0.8	1.3 ± 0.1	2.7 ± 0.2	0.1 ± 0.9	0.7 ± 0.3	0.9 ± 0.2	1.1 ± 0.2	0.6 ± 1	2 ± 0.3	0.1 ± 0.9
17:0	10.1 ± 0.3	21.8 ± 1	13.9 ± 0.1	9.7 ± 0.2	14.9 ± 0.1	14.2 ± 0.2	10.7 ± 0.2	6.4 ± 0	15.9 ± 0.7	28.5 ± 0.2	3.2 ± 0.1
18:1ω9c	2 ± 0.1	2.4 ± 0.7	2.2 ± 0.1	3.9 ± 0.2	1.1 ± 0.1	1.7 ± 0.2	3.1 ± 0.3	2.1 ± 0	2.3 ± 0.7	1 ± 0.2	1.4 ± 0.4
18:1ω7c	4.1 ± 0.2	10.8 ± 0.9	3.8 ± 0.1	11.9 ± 0.2	1.4 ± 0.1	1.3 ± 0.2	2.8 ± 0.2	1.6 ± 0	2.7 ± 0.7	5.2 ± 0.1	4.5 ± 0.2
18:0	0.9 ± 0.2	1.6 ± 0.9	0.8 ± 0.1	0.9 ± 0.2	2.6 ± 0.1	1.4 ± 0.2	1.4 ± 0.2	1 ± 0.1	2 ± 0.7	0.8 ± 0.1	0.8 ± 0.1
19:1a	0.7 ± 0.2	0.5 ± 0.6	0.3 ± 0.1	0.5 ± 0.1	0 ± 0	0.5 ± 0.3	0.6 ± 0.4	1 ± 0.1	0.3 ± 0.9	0.3 ± 0.9	0 ± 0.9
Sum SFA	71.3 ± 0.2	71 ± 0.6	71.9 ± 0.1	54.1 ± 0.2	88.9 ± 0	84.8 ± 0.2	76.3 ± 0.2	84.8 ± 0.1	88.8 ± 0.5	77.3 ± 0.1	76.4 ± 0.1
Sum MUFA	15.2 ± 0.1	20.2 ± 0.6	10.2 ± 0.1	23.6 ± 0.2	3.4 ± 0.1	6.6 ± 0.1	11.4 ± 0.2	10.6 ± 0	8.7 ± 0.5	10.2 ± 0.1	10 ± 0.1

The qualitative properties of the thraustochytrid residual oil for potential biodiesel application, based on the fatty acids profiles after winterization, are reported in Table 5. The saponification values ranged from 109 to 183, showing disparity of the average fatty acid chain length of the British strains. The iodine values ranged from 3 to 21, showing different oxidative stability profiles. For instance, the high iodine value of strain OL5TA (21) and SW4T2B (18) could be explained by the high level of MUFA (23.6 and 20.2% of TFA, respectively), particularly 18:1ω7c, thus reflecting a richness in double bonds of the oil. On the other hand, SW7T4C1 and SW7T4C6 showed a lower iodine value (3 and 6, respectively) due to their lower levels of 18:1ω7c (1.4 and 1.3% of TFA, respectively) and high levels of SFA (88.9 and 84.8% of TFA, respectively). This feature is confirmed by the value for the degree of unsaturation, which is directly proportional to the relative level of unsaturated fatty acids. Good ignition properties are achieved with a fatty acid profile rich in SC-SFA, which implies low iodine and saponification values and therefore a high cetane number. The strains SW4T2B, OL5TA and SM01 exhibited the highest cetane numbers (90, 91 and 93, respectively). Nonetheless, the remaining strains still showed a high cetane number ranging from 73 to 86. The LCSF is a good indicator of the richness of an oil in LC-SFA, which are undesirable for biodiesel as they have high melting points. Indeed, for biodiesel application, the oil must remain liquid at low temperatures to avoid clogging of engines and fuel lines. Thus, a low LCSF value will result in a low CFPP, which is a good intrinsic property for biodiesel. In the present study, strains SW4T2A, SW4T8A, OL5TA and SM01 showed very low CFPP values of −9.8 °C or below, while strains SW7T4C1 and TL18 showed the highest CFPP of 0.4 and −0.5 °C, respectively.

**Table 5:** Qualitative properties of biodiesel for ten British thraustochytrid strains and reference strain *Sicyoidochytrium* sp. based on their fatty acid profiles in Table 4

Strain	SW4T2A	SW4T2B	SW4T8A	OL5TA	SW7T4C1	SW7T4C6	SW7T7C	SW7T8C	TL18	SM01	102979
SV	183	114	131	109	154	156	149	182	159	111	159
IV	14	18	9	21	3	6	10	9	8	9	9
CN	73	90	86	91	81	80	81	74	79	93	78
DU	30	39	20	46	6	13	22	21	17	20	20
LCSF	2.0	3.8	1.8	1.8	5.4	3.5	3.0	2.4	5.1	2.1	6.8
CFPP	−10.1	−4.5	−10.8	−10.9	0.4	−5.5	−6.9	−9.0	−0.5	−9.8	4.9

*SV* saponification value is correlated with the average chain length (molecular weight) of the fatty acids profile, *IV* the iodine value represents the oxidative stability by evaluating the total unsaturation of the fatty acid profile, *CN* the cetane number represents the ignition property, *DU* denotes the degree of unsaturation of the fatty acids which represents the oxidative stability during long term storage, *LCSF* a high long-chain saturation factor indicates an oil rich in LC-SFA and therefore more sensitive to crystallization, *CFPP* (°C) the cold filter plugging point is the temperature at which the biodiesel tends to crystallize which can clog the filters and fuel lines (Ruangsomboon 2015)

As shown in Table 6, British thraustochytrids under the growth conditions used in this study, and after a winterization process, could meet the reference standard criteria for biodiesel application according to the European Standard EN 14214 and ASTM International standard D6751. All of their cetane numbers are above 51 and iodine values below 120, mainly due to their high SFA composition. In comparison, Byreddy et al. (2015), in a similar sub-study on biodiesel, found that full crude oil extracted from *Schizochytrium* sp. S31 and *Thraustochytrium* sp. AMCQS5-5 did not meet the biodiesel standard requirement due to the high relative level of LC-PUFA contained in both strains. Nonetheless, *Schizochytrium* sp. S056 almost met the EN 14214 standard (Chen et al. 2015), showing that thraustochytrid crude oil rich in palmitic acid could potentially be used for biodiesel exploitation directly without requiring a winterization step. However, the CFPP (7.5 °C) was higher when compared to the findings of this study (as low as −10.9 °C) which would be a disadvantage (Table 6). Crude oil from other microorganisms, such as phototrophic microalgae and cyanobacteria, also have shown potential for biodiesel application with low CFPP values, but higher SV and DU values were observed, suggesting higher content in LC-PUFA, thus showing the importance of the winterization step to remove these (Francisco et al. 2010; Da Rós et al. 2013). Lee Chang et al. (2015) showed that thraustochytrid cultivation for hydroprocessed biodiesel production could be viable using agro-industrial by-products (e.g. molasses or glycerol) as sole carbon source. However, transesterified biodiesel had a low-energy return on energy invested factor of 0.43 (meaning that for 1 unit of energy produced, 2.32 units of energy are needed). As a result, additional research would still be required to improve growth conditions, fatty acid profile and yield for biodiesel production from thraustochytrids. The most promising way forward may rather see a primary objective of optimizing PUFA production by thraustochytrids with by-product oil lacking PUFA also being available for biodiesel application.

**Table 6:** Intrinsic properties calculated from fatty acid profiles of oils from thraustochytrids and other sources for potential biodiesel application

	CN	IV	SV	CFPP	DU	LCSF	References
EN 14214	>51	<120		–	–	–	
ASTM D6751	>47	–		–	–	–	
*Schizochytrium sp.* S056	49	118		7.5			Chen et al. (2015)
*Schizochytrium sp.* S31	47	99	234	4.5	63	3.8	Byreddy et al. (2015)
*Thraustochytrium sp*. AMCQS5-5	44	157	165	1.9	70	5.8	Byreddy et al. (2015)
British thraustochytrid OL5TA	91	21	109	−10.9	46	1.8	This study
British thraustochytrid SM01	93	9	111	−9.8	20	2.1	This study
*Chlorella*	57	65	225	−4.6	71	3.8	Francisco et al. (2010)
*Phaeodactylum*	56	59	217	−12.3	53	1.3	Francisco et al. (2010)
Sunflower	50	132	(193)	−3	–	–	Ramos et al. (2009) (Gopinath et al. 2009)
Palm	61	57	(205)	10	–	–	Ramos et al. (2009) (Gopinath et al. 2009)
*Microcystis aeruginosa* NPCD-1	–	57	210		61	5.7	Da Rós et al. (2013)
*Trichormus* sp.CENA77	–	68	213		70	5	Da Rós et al. (2013)

Abbreviations: see Table 5

## Conclusion

This initial screening study of new British thraustochytrid strains, based on their fatty acid profiles following growth in low-glucose and complex nitrogen source-rich medium MCBHB, showed that they have potential for DHA production, particularly strains TL18 and SW7T4C1 (highest culture DHA concentration) and strain OL5TA (highest relative proportion of TFA as DHA). Further work is now required to optimize culture conditions (including further investigation of different carbon and nitrogen sources in the medium) to increase the biomass and fatty acid yields of these British strains in order to generate competitive levels of production. In addition, a mathematical analysis of residual fatty acids after a theoretical winterization step revealed that the by-product oil could be potentially suitable for biodiesel production.
